# The Seedlings of Different *Japonica* Rice Varieties Exhibit Differ Physiological Properties to Modulate Plant Survival Rates under Submergence Stress

**DOI:** 10.3390/plants9080982

**Published:** 2020-08-03

**Authors:** Yu-Syuan Li, Shang-Ling Ou, Chin-Ying Yang

**Affiliations:** Department of Agronomy, National Chung Hsing University, Taichung 40227, Taiwan; shaneimayday@gmail.com (Y.-S.L.); slou@dragon.nchu.edu.tw (S.-L.O.)

**Keywords:** *Oryza sativa*, Submergence, Activity of antioxidant enzymes, Chlorophyll content

## Abstract

*Oryza sativa* is a major food crop in Asia. In recent years, typhoons and sudden downpours have caused field flooding, which has resulted in serious harm to the production of rice. In this study, our data revealed that the plant heights of the five *Japonica* varieties increased during submergence. The elongation rates of TN14, KH139, and TK9 increased significantly during submergence. Chlorophyll contents of the five varieties significantly decreased after submergence and increased after recovery. Moreover, the chlorophyll content of KH139 was significantly higher than those of the other four varieties after recovery. The plant survival rates of the five varieties were higher than 50% after four-day submergence. After eight-day submergence, the survival rate of KH139 remained at 90%, which was the highest among the different varieties. The KH139 presented lower accumulation of hydrogen peroxide and the catalase activity than those of the other four varieties under submergence. The *sucrose synthase 1* and *alcohol dehydrogenase 1* were induced in KH139 under submergence. The results presented that different varieties of *japonica* rice have different flood tolerances, especially KH139 under submergence was superior to that of the other four varieties. These results can provide crucial information for future research on *japonica* rice under flooding stress.

## 1. Background

Global climate change has led to extreme climate in recent years. Global warming has caused sea-level rise, affected crop cultivation, and led to decreased crop production. The frequency of flooding disasters worldwide caused by biological stresses, such as high temperature, low temperature, droughts, and salt-damaged soils caused by extreme climates, has increased by approximately 65% in the past 25 years [1]. *Oryza sativa* is a major food crop worldwide and it is widely cultivated in Asia. Flooding disasters have considerably affected agricultural production in Asia, particularly in several Southeast Asian countries, such as the Philippines, Myanmar, and Indonesia, and have led to a considerable decrease in crop yield. Thus, flooding disasters have become an issue that must be urgently confronted [2].

In a normal growth environment, higher plants perform aerobic respiration and transport the products of photosynthesis from the source to the sink through the glycolysis pathway, so that the related reactions of carbon metabolism can occur [3]. Sucrose can be decomposed into glucose and fructose in two ways. Sucrose can be catalyzed into fructose and glucose through sucrose invertase, or it can be catalyzed into fructose and uracil-diphosphate glucose through sucrose synthase (SUS). When hypoxia is caused by flooding stress, a plant limits the pathway of sucrose invertase, reduces energy consumption, and increases SUS activity for preserving the source of reactants that are essential for glycolysis [4]. Research has indicated that during flooding treatment, the expression of the *SUS* genes of rice increases with the flooding period. Moreover, ethylene induces submergence1 (*SUB1*), which regulates the expression of *SUS* genes [5]. The expression of *SUS* genes increases when corn is subjected to hypoxia treatment. Corn under hypoxic stress is speculated to induce sucrose for accelerated decomposition in order to maintain the glycolysis reaction and reduce the ATP consumption [6]. In addition, research have shown that plants lacking genes that required for fermentation, such as *alcohol dehydrogenase* (*ADH*) and *pyruvate decarboxylase* (*PDC*), or mutants defective in sucrose metabolism, such as *SUS*, revealed lower tolerance in hypoxia condition [7]. When crops are fully submerged, the gas exchange between plants and air significantly decreases. Gases, such as O_2_ (molecular oxygen), CO_2_ (carbon dioxide), and ethylene, spread very slowly in water, preventing effective exchange and causing hypoxia in the plant’s root, stem and leaves, which causes hypoxic stress [8]. Because the aerobic respiration of the rice plant is restricted under flooding stress, the plant performs anaerobic respiration and enters the fermentation route, which leads to the accumulation of toxic substances, such as ethanol and lactic acid, in the cells. The accumulation of ethanol damages the cell membrane and the membrane structure of the organelle. Lactic acid acidifies the cytoplasm, affects enzyme action in crucial metabolic pathways, and influences crop viability in a hypoxic environment [9].

Reactive oxygen species (ROS) also play a major role when the plants are under hypoxic stress. ROS are produced through induction and cause oxidative stress inside the plant cell [10,11]. Some ROS include the superoxide anion (O_2_^−^), H_2_O_2_, and the hydroxyl radical (OH). The presence of excessive free radicals damages the DNA or RNA in the cells or causes protein or lipid peroxidation. Under hypoxic stress, the content of H_2_O_2_ as well as the gene expressions of *ascorbate peroxidase* (*APX*) and *superoxide dismutase* (*SOD*) in grape sprouts increase significantly, which indicates that the activated oxygen group can participate in the regulation of antioxidant enzyme transcription [12]. Studies have indicated that the H_2_O_2_ in ROS can act as a signaling molecule to participate in the regulation of the reaction, growth, and development of plants under environmental stress [13].

Under flooding stress, FR13A lowland rice and deepwater rice exhibit two adaptation strategies. The quiescence strategy is one strategy. The accumulation of ethylene in the plant cells of FR13A lowland rice increases under flooding stress. The accumulation of ethylene induced the expression of the ethylene transcription factor submergence 1A (*SUB1A*) for downstream signal regulation. SUB1A influence gibberellin signal transduction and the metabolic pathway of carbohydrate are affected, which leads to a two-week stillness of FR13A lowland rice under flooding stress. The rice growth becomes normal again after the water levels recede [14]. The other strategy is the escape strategy. The accumulation of ethylene inside the plant cells of deepwater rice under flooding stress causes the ethylene transcription factors Snorkel1 (SK1) and Snorkel2 (SK2) to directly or indirectly regulate the synthetic pathway and signal transduction process of gibberellin. Thus, the above-ground parts of rice are elongated and further protrude from the water to perform gas exchange [15]. Ethylene accumulation is also found within the plant of the rice varieties without *SK1*, *SK2*, and *SUB1A* under flooding stress. This accumulation regulates gibberellin signal transduction and the metabolic pathway of carbohydrate, such that the above-ground parts may protrude from the water to perform gas exchange. However, because most of its energy is consumed, the plant often cannot protrude from the water in time. Thus, it displays the physiognomic traits of withering, yellowing, and even death. These traits indicate the occurrence of low-oxygen-escape syndrome (LOES) [4].

Numerous studies have examined the physiological and molecular reaction mechanisms of flood-resistant rice with *SUB1* locus in a fully submerged environment. However, the physiological and molecular performance of the commonly cultivated varieties of *japonica* rice under flooding stress has rarely been investigated. Therefore, five varieties of good-quality *japonica* rice that are cultivated in Taiwan were subjected to submergence experiments and plant survival rate tests. Moreover, their total chlorophyll contents and antioxidant enzyme activities were determined. The physiological and molecular reactions of the seedlings of the *japonica* rice varieties were investigated under submergence conditions. Thus, crucial information was obtained on the properties of the aforementioned rice varieties under flooding stress.

## 2. Results

### 2.1. Plant Heights, Dry and Fresh Weights of the Five Varieties of Japonica Rice Under Diverse Submergence Periods

**Figure 1:**
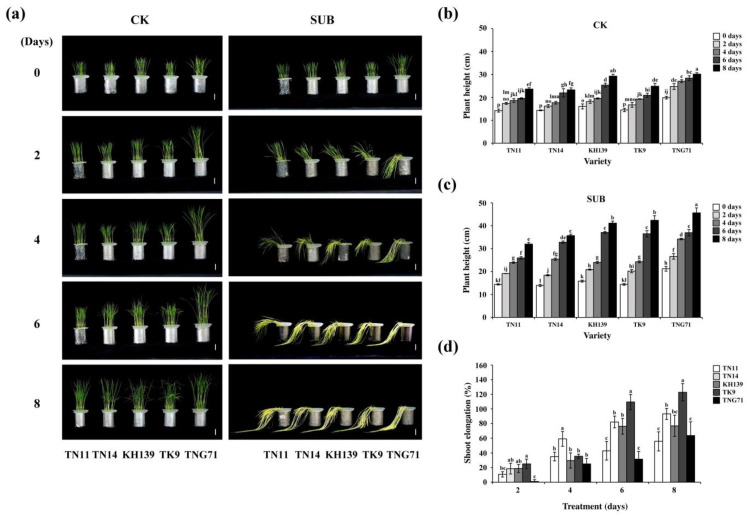
Characterization of the *japonica* rice varieties seedlings grown during submergence stress. (**a**) Photographs of nine-day-old rice seedlings after full-submergence treatment for 0, two, four, six, and eight days; (**b**) plant heights of the nine-day-old seedlings following normal growth for 0, two, four, six, and eight days; (**c**) plant heights of the nine-day-old seedlings subjected to full-submergence treatment for 0, two, four, six, and eight days; and, (**d**) elongation rates of the above-ground parts of the nine-day-old seedlings subjected to full-submergence treatment for two, four, six, and eight days. CK (control check) was the control group; SUB (submergence) was the full-submergence treatment group; and scale bar = 5 cm. Statistical Analysis System (SAS) 9.4 was used to conduct Duncan’s analysis. The different alphabets in (**a**,**b**) indicate the significant differencesin the number of treatment days and performance among the five rice varieties (*p* < 0.05). The different alphabets in (**c**) indicate the significant difference between the performance of the rice varieties (*p* < 0.05). Each treatment involved at least 30 plants, and independent experiments were repeated more than three times.

Under flooding stress, varieties of rice that are not flood-resistant exhibit LOES. Their petioles, stems, and leaves elongate as the flooding treatment period increase [4]. Thus, five varieties of good-quality *japonica* rice commonly grown in Taiwan were subjected to different tests to understand the physiological traits of *japonica* rice varieties under diverse flooding periods. The five varieties used in this study were Tainan 11 (TN11), Tainan 14 (TN14), Kaohsiung 139 (KH139), Taiken 9 (TK9), and Tainung 71 (TNG71). The 9-day-old seedlings were subjected to full-submergence treatments for 0, two, four, six, and eight days and compared with the control group without treatment. The changes in the heights of the plants as well as the dry and fresh weights of their above-ground parts were measured after the aforementioned periods. Following two-day submergence treatment, TNG71 exhibited complete lodging and TN11, TN14, KH139, and TK9 exhibited half-lodging. On the eighth day of the submergence treatment, the five rice varieties exhibited the physiognomic traits of significant withering and yellowing, slender leaves, and lodging (Figure 1). The results indicated that the plant heights of the five varieties increased as the flooding treatment period increased (Figure 1b,c). The plant elongation rates of TN14, KH139, and TK9 increased significantly (by 82.09%, 76.18%, and 109.51%, respectively) on the sixth day of the submergence treatment (Figure 1d).

**Figure 2:**
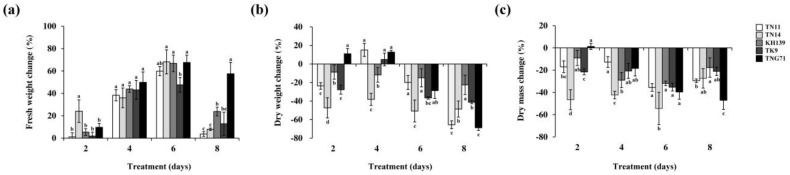
Comparison of fresh, dry weight, and ratio of dry weight to fresh weight in *japonica* rice varieties for different submergence periods. (**a**) The fresh weights, (**b**) dry weights and (**c**) ratio of dry weight to fresh weight of nine-day-old seedlings subjected to full-submergence treatment for two, four, six, and eight days compared with those of the control group. SAS 9.4 was used to conduct Duncan’s analysis. The different alphabets inidacte significant difference in the performance of different varieties for the same number of treatment days (*p* < 0.05). Each treatment involved at least 30 plants, and independent experiments were repeated more than three times.

To explore the effects of submergence stress on the dry matter losses of the five *japonica* rice varieties, nine-day-old seedlings of TN11, TN14, KH139, TK9, and TNG71 were subjected to full-submergence treatments for 0, two, four, six, and eight days. After the treatment, the fresh weight, dry weight, and ratio of dry weight to fresh weight of the above-ground part were determined for the plants of the five rice varieties (Table 1). The results indicated that the fresh weight of the above-ground part of TN14 following two-day full-submergence treatment was 36% higher than that of the control group. This increase in the fresh weight was significantly higher than those of the other four varieties. No significant differences were observed in the fresh weights following four- and six-day full-submergence treatments. Following 8-day full-submergence treatment, the fresh weight of TNG71 increased by 57.5%, which was significantly higher than the fresh weight increased for the other four varieties (3.7%, 7.9%, 23.7%, and 12.8%) (Figure 2a). Following eight-day full-submergence treatment, the dry weight of KH139 was 22.6% lower than that of the control group. However, the dry weight of KH139 was higher than those of the other four varieties (65.6%, 48.7%, 41.4%, and 68.9% lower than that of the control group) (Figure 2b). Following eight-day full-submergence treatment, the dry mass change of TNG71 was 47% lower than that of the control group. The dry mass change of TNG71 was significantly lower than those of the other four varieties (whose decreases in percentage of dry mass change were 29.6%, 27.4%, 17.7%, and 21.1%) (Figure 2c). Thus, the dry matter loss of TNG71 following eight-day full-submergence treatment was higher than those of the other four varieties.

### 2.2. Changes in the Chlorophyll Contents and Survival Rates in the Five Varieties of Japonica Rice Under Submergence Stress

**Figure 3:**
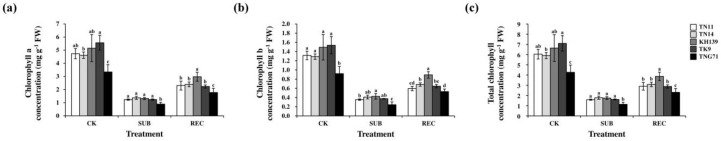
The chlorophyll contents of five *japonica* rice varieties for different submergence periods. The measured chlorophyll a, chlorophyll b, and total chlorophyll contents of the above-ground parts of nine-day-old seedlings subjected to full-submergence treatment for 8 days followed by 7-day recovery. (**a**) Determination of the chlorophyll a content. (**b**) Determination of the chlorophyll b content. (**c**) Determination of the total chlorophyll content. CK (control check) was the control group, SUB (submergence) was the full-submergence treatment group, and REC (recovery) was the recovery group. SAS 9.4 was used to conduct Duncan’s analysis. The different alphabets indicatea significant difference in the performances of the various rice varieties for the same number of treatment days (*p* < 0.05). Independent experiments were repeated more than three times.

The chlorophyll a, chlorophyll b, and total chlorophyll contents of the above-ground parts of TN11, TN14, KH139, TK9, and TNG71 were measured following eight-day full-submergence treatment to explore the changes in the chlorophyll contents of the five *japonica* rice varieties under submergence stress. The experimental results indicated that the chlorophyll a, b, and total chlorophyll contents of the five rice varieties significantly decreased under flooding stress and increased following seven-day recovery after the flood. TK9 exhibited the highest rates of decline in the chlorophyll a, b, and total chlorophyll contents under submergence stress (78%, 76%, and 77%, respectively). TN14 exhibited the lowest rates of decline in the chlorophyll a, b, and total chlorophyll contents under flooding stress (70%, 68%, and 70%, respectively). KH139 exhibited the highest rates of increase in the chlorophyll a and total chlorophyll contents (126% and 121%, respectively) after recovery. TNG71 displayed the highest rate of increase in the chlorophyll b content (121%) after recovery. It is worth noting that TN14 also exhibited the lowest rates of increase in the chlorophyll a, b, and total chlorophyll contents (75%, 66%, and 73%, respectively) after recovery (Figure 3).

**Figure 4:**
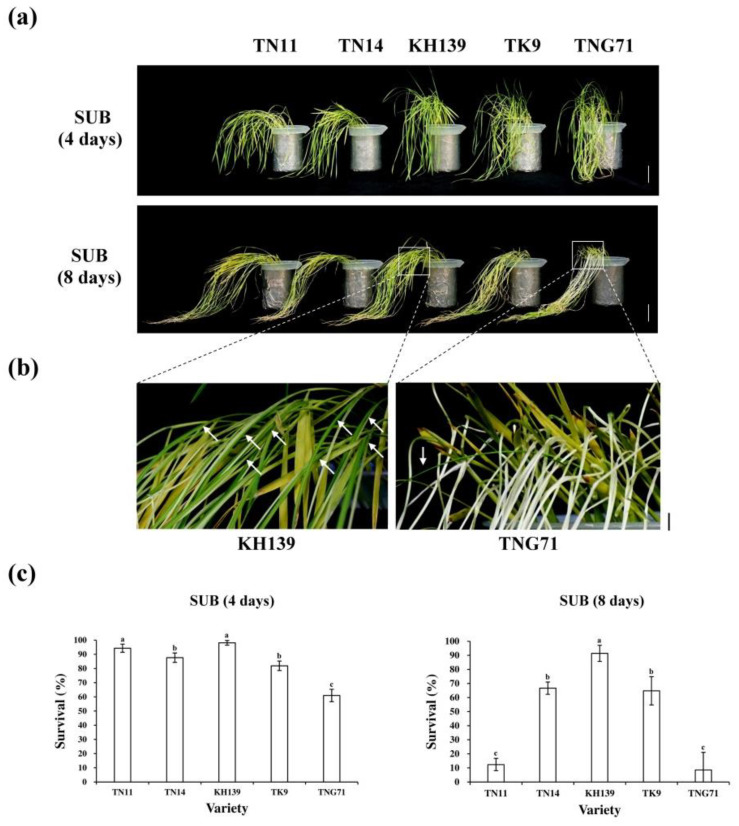
Characterization and survival rates of the five *japonica* rice varieties after recovery following different submergence periods. (**a**) Photographs of 9-day-old seedlings subjected to full-submergence treatment for four and eight days followed by seven-day recovery (bar = 5 cm); (**b**) Photographs of enlarged images of KH139 and TNG71 (bar = 1 cm); and (**c**) survival rates of 9-day-old seedlings subjected to full-submergence treatment for four and eight days followed by seven-day recovery. SUB (submergence) was the full-submergence treatment group. SAS 9.4 was used to conduct Duncan’s analysis. The different alphabets indicate significant differences in the performance of the different rice varieties (*p* < 0.05). Each treatment involved at least 35 plants, and independent experiments were repeated more than three times.

Studies indicate that submergence-tolerant lowland rice varieties contain *SUB1A-1* genes conferring flooding stress tolerance [5]. The plant survival rates of the rice varieties were examined under flooding stress due to the absence of *SUB1A* genes in the five varieties of *japonica* rice considered in this study. The results indicated that the plant survival rates of TN11, TN14, KH139, TK9, and TNG71 following four-day full-submergence treatment were 94.3%, 87.6%, 98.1%, 81.9%, and 61.0%, respectively. The plant survival rates of the aforementioned varieties following full-submergence treatment for eight-day were 12.4%, 66.7%, 91.4%, 64.8%, and 8.6%, respectively (Figure 4a,b). The results indicate that the plant survival rates of the five varieties following a four-day flood were higher than 50%. Following an eight-day flood, KH139 had the highest plant survival rate, which remained at 90%. The plant survival rates of TN11 and TNG71 were less than 15%.

### 2.3. H_2_O_2_ Accumulation and Antioxidant Enzyme Activities of the Five Varieties of Japonica Rice Under Submergence Stress

**Figure 5:**
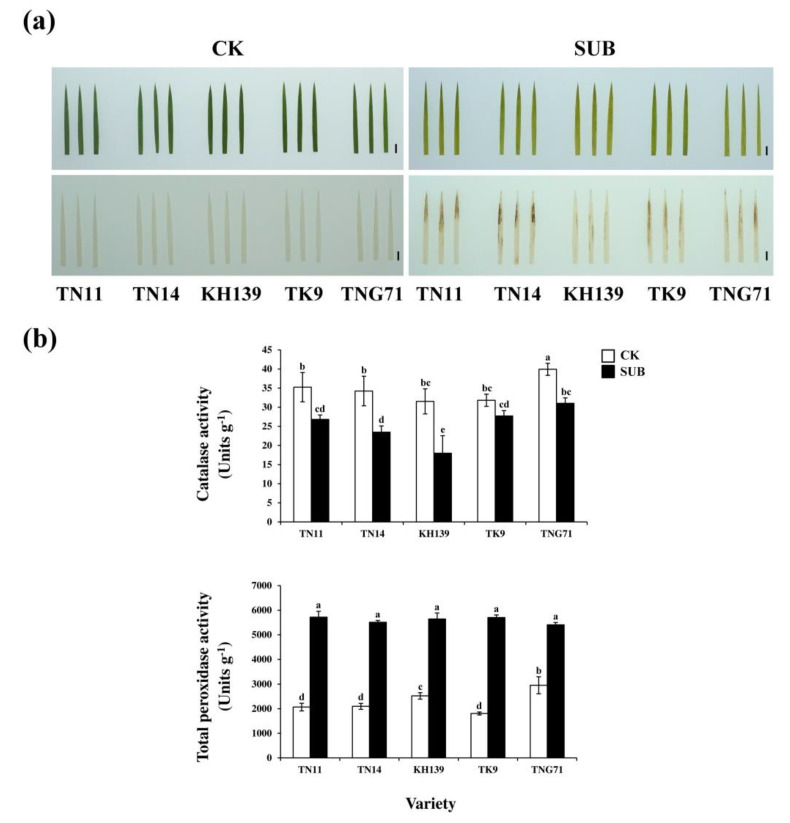
H_2_O_2_ accumulation and activities of antioxidative enzymes of the *japonica* rice varieties under submergence stress. (**a**) The upper panel of pictures showed the leaves before DAB staining. The down panel of pictures showed the DAB (3,3′-diaminobenzidine) staining of H_2_O_2_ in nine-day-old seedlings subjected to eight-day full-submergence treatment (bar = 1 cm). (**b**) Determination of the antioxidant enzyme activities of 9-day-old seedlings subjected to eight-day submergence treatment. CK (control check) was the control group, SUB (submergence) was the full-submergence treatment group, SAS 9.4 was used to conduct Duncan’s analysis, The different alphabets indicate significant differencesin the performance of the five rice varieties (*p* < 0.05). Independent experiments were repeated more than three times.

To understand the H_2_O_2_ accumulation in the five varieties under submergence stress, nine-day-old seedlings of TN11, TN14, KH139, TK9, and TNG71 were subjected to eight-day full-submergence treatment, followed by 3, 3′-diaminobenzidine (DAB) staining on the second leaf of each plant. The experiment results indicated that in the control group, the second leaves of TN11, TN14, KH139, TK9, and TNG71 did not appear reddish brown. After full-submergence treatment, all of the second leaves of the aforementioned five varieties appeared reddish brown. A large quantity of H_2_O_2_ accumulated in the leaf tips of TN11 and leaf margins of TN14. Such accumulation was also found in the leaf margins and bodies of TK9 and TNG71. The H_2_O_2_ accumulation of KH139 was significantly lower than that of the other four rice varieties (Figure 5a).

Analysis of the antioxidant enzyme activity revealed that the catalase (CAT) activities of the five rice varieties under full-submergence stress decreased to a greater extent than those of the control group, with the CAT activity of KH139 exhibiting the largest decrease. Analysis of the total peroxidase (POD) activity revealed that the POD activities of KH139 and TNG71 in the control group were significantly higher than those of TN11, TN14, and TK9. The POD activities of the five rice varieties under full-submergence stress exhibited a considerably larger increase than those of the control group (Figure 5b).

### 2.4. Anaerobic-respiration-related Gene Expressions in the Five Varieties of Japonica Rice Seedlings Under Submergence Stress

**Figure 6:**
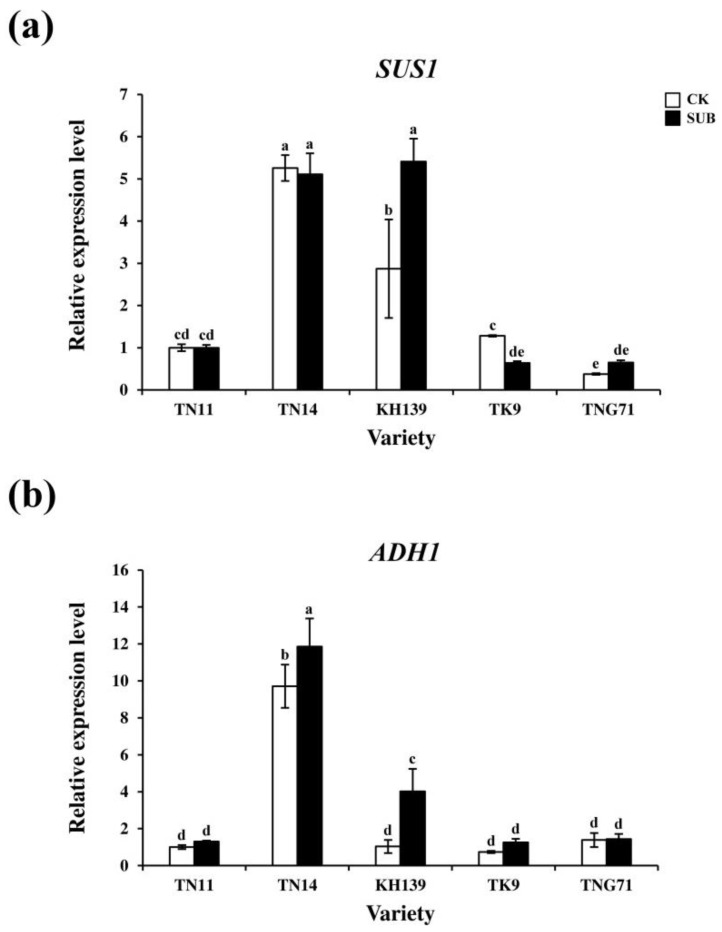
Transcript levels of hypoxia-related genes for the *japonica* rice varieties seedlings under submergence stress. 9-day-old seedlings were subjected to full-submergence treatment for two days, and their above-ground parts were then subjected to qRT-PCR analysis. (**a**) Expressions of *SUS1* of glycolysis-related genes and (**b**) *ADH1* of alcoholic fermentation-related genes. CK (control check) was the control group, and SUB (submergence) was the full-submergence treatment group. SAS 9.4 was used to conduct Duncan’s analysis. The different alphabets indicate significant differencesin the performance of the five rice varieties (*p* < 0.05). Independent experiments were repeated four times.

Nine-day-old seedlings of TN11, TN14, KH139, TK9, and TNG71 were subjected to full-submergence treatment for two days in order to examine the anaerobic-respiration-related gene expressions of the five varieties of *japonica* rice under submergence stress. The seedlings were then tested for the gene expressions of sucrose synthase 1 (*SUS1*) and alcohol dehydrogenase 1 (*ADH1*). The results indicated that, in the control group, the *SUS1* expressions of TN14 and KH139 were higher than those of TN11, TK9, and TNG71. The *SUS1* expression of KH139 that underwent submergence treatment increased to a considerably greater extent than those of the control group (Figure 6a). In the control group, the *ADH1* expression of TN14 was higher than those of TN11, KH139, TK9, and TNG71. The *ADH1* expressions of TN14 and KH139 that underwent submergence treatment decreased to a considerably greater extent than those of the control group (Figure 6b). The results indicated that the expressions of *SUS1* and *ADH1* were induced in the KH139 plant under submergence stress.

## 3. Discussion

Rice is the main food crop in Asia. Typhoons and sudden downpours are the major causes of agriculture-related disasters in Taiwan. The aforementioned natural disasters cause field flooding, severe harm to rice cultivation, and considerable losses in rice production. Under flooding stress, the oxygen that is essential for crops cannot be effectively exchanged in water, which causes hypoxic stress. The ability of crops to perform photosynthesis is impeded in a hypoxic environment, which causes problems such as leaf yellowing, impeded root system growth, and decreased mitochondrion metabolism. The cultivation of direct-seeded rice has become common in Asia in recent years. When compared with transplanted cultivation, direct seeding can considerably reduce field water consumption and labor as well as increase productivity. Direct-seeded rice can be divided into dry-, wet-, and water-seeded rice [16]. Water-seeded rice has the best emergence effect among the aforementioned three rice types; however, the cultivation of water-seeded rice involves difficulties that are related to the emergence of its seedlings in flooded fields or the survival of its seedlings after emergence, especially when the seeds fall into deep soil layers. Thus, the rate of successful cultivation of direct-seeded rice can be increased by selecting seedlings of rice varieties with high flood tolerance [17].

Research on varieties of rice that are not flood-resistant indicates that flooding stress causes an increase in the ethylene concentration of the plant tissue due to hypoxia and it enhances the gene expression of *SUB1C*. Moreover, it induces GA downstream gene expression, which causes the elongation of the petioles, stems, and leaves of rice, as well as LOES [4]. Our experimental results indicated that the plant heights of TN11, TN14, KH139, TK9, and TNG71 increased with the submergence treatment period (Figure 1). The plant elongation rates of TN14, KH139, and TK9 significantly increased on the sixth day of the flooding treatment (Figure 1). Thus, the above-ground parts of the five rice varieties were elongated under flooding stress, which indicated that the five rice varieties experienced LOES under submergence stress. Under flooding stress, chloroplasts can cause damage and reduce the photosynthesis efficiency in plants [18]. In addition, the accumulation of ethylene in rice varieties that are not flood-resistant causes the elongation of their above-ground parts, which results in the consumption of carbohydrate in the plant, photo damage to the plant, and degradation of the chlorophyll content in the leaves during flooding stress [19,20]. Stresses lead to the accumulation of excessive activated oxygen groups, such as H_2_O_2_ and O_2_^−^, in the leaves of plants, which causes issues, such as oxidative stress and lipid peroxidation in the plants [21,22]. Our results revealed that the chlorophyll a, chlorophyll b, and total chlorophyll contents of the five varieties of *japonica* rice decreased significantly following eight-day full-submergence treatment. However, the values of the aforementioned parameters increased significantly following seven-day recovery after the full-submergence treatment. The chlorophyll content of KH139 was significantly higher than those of the other four varieties (Figure 3), which indicated that the cells of KH139 were less harmed by flooding stress than those of the other four varieties were and that KH139 returned to normal growth faster than the other four varieties did. Moreover, the results of DAB staining indicated that a low amount of H_2_O_2_ accumulated in KH139 under submergence stress (Figure 5a). Biotic or abiotic stresses cause the accumulation of activated oxygen groups inside the plant cells. To preserve the oxidation equilibrium inside their cells, plants induce the antioxidant enzyme system to remove the activated oxygen groups for preventing these groups from damaging their cells and for enhancing their stress tolerance [23,24,25]. Analysis of the antioxidant enzyme activity revealed that the CAT activity of KH139 was significantly lower than those of the other four varieties, probably due to its low H_2_O_2_ accumulation under submergence stress. Under submergence stress, no significant difference was noted in the total POD activities of KH139 and the other four rice varieties (Figure 5b). The genes *SUS1* and *ADH1* were induced in the plants of KH139 under submergence stress, and KH139 had higher plant survival rates following eight-day submergence treatment (Figure 4 and Figure 6). These results indicated that the survival rate of KH139 might increase under flooding stress due to the enhancement of its hypoxia-related gene expression.

The seedlings of the five varieties exhibited the physiognomic traits of withering, yellowing, slender leaves, and lodging under flooding stress. However, four days after the flooding treatment, the plant survival rates of the five rice varieties were higher than 50%. Excluding TNG71, the plant survival rates of the other four rice varieties were 80% or higher, which indicated that, although *japonica* rice varieties do not contain *SUB1A*, their plant survival rates remain high if the flooding period is short. Moreover, following eight-day submergence treatment, the plant survival rates of three varieties, namely TN14, KH139, and TK9, were 60% or higher (Figure 4). Previous scholars have demonstrated that the *SUB1A* gene is a crucial response gene for rice plants to become flood-resistant through ethylene signaling. The *Indica* rice cultivars, such as FR13A, which contains *SUB1A* revealed extremely submergence tolerant; 100% of 10-day-old seedlings survived 7 days of complete submergence [26]. In addition to *SUB1A*, this study indicated that other crucial reaction mechanisms regulate plant flood tolerance in rice varieties that do not comprise this gene.

## 4. Conclusions

The changes in the plant heights of five *japonica* rice varieties after full-submergence treatment indicated that the chlorophyll content of the plants decreased significantly and the plants experienced LOES under flooding stress. However, the accumulation of H_2_O_2_ in the leaves of the five varieties and their peroxidase activities were different. Moreover, molecular-level analysis indicated that the expressions of *SUS1* and *ADH1*, which were induced by hypoxia signaling, were different, which suggested that different varieties of *japonica* rice have different flood tolerances. In this study, we recommend KH139 as a good potential breeding material for flood tolerance when compared with the other four *japonica* varieties of rice seedlings. These results can provide crucial information for future research on *japonica* rice under flooding stress and on direct-seeded rice.

## 5. Materials and Methods

### 5.1. Plant Materials and Growth Conditions

Five good-quality *japonica* rice varieties that are commonly planted in Taiwan, namely Tainan 11 (TN11), Kaohsiung 139 (KH139), Taiken 9 (TK9), Tainung 71 (TNG71), and Tainan 14 (TN14) were selected for experiments. These rice plants were irrigated lowland rice. Their seeds were cleaned and disinfected with 3% sodium hypochlorite for 30 min, followed by cleaning with sterile deionized water three times for completely removing the sodium hypochlorite residue and introducing sterile deionized water. The seeds were then placed in a growth box at 28 °C with lighting cycles of 16 h of light and eight hours of darkness (the brightness was 36 μmol m^−2^s^−1^). The seeds that germinated after 2-day wet cultivation were placed on iron grids in 500-mL beakers wrapped with aluminum foil and then cultivated with the Kimura B solution [27]. A total of 35 to 40 seedlings were cultivated in each beaker. The hydroponic growing medium was renewed every two days until the seedlings were nine days old. Subsequently, experiments and treatments were performed. Flooding treatment was conducted by placing nine-day-old seedlings in a water tank (40-cm long, 40-cm wide, and 60-cm high) with a water level of 55 cm (with this water level, plants do not protrude from the water following eight-day flooding treatment) for 0, two, four, six, and eight days. In the growth box, the temperature was 28 °C, the lighting alternated between 16 h of light and eight hours of darkness, and the brightness was 236 μmol m^−2^s^−1^. The physiognomic traits of the plants were investigated, and the plant materials were subjected to further analyses.

### 5.2. Seedlings Plant High, Fresh Weight, Dry Weight Chlorophyll Content and Survival Rate Determination

The nine-day-old seedlings were subjected to full-submergence treatments for 0, two, four, six, and eight days. The above-ground heights of their plants were measured after the aforementioned full-submergence treatment durations. For plant height measurement, the above-ground height of the plant was defined as the distance between the base of a straightened seedling and the tip of its longest leaf. In each independent experiment, the mean heights of at least 30 plants were measured. The experiments were repeated at least three times. The plant elongation rates were calculated using the technique of Zhu et al. [28]. The plant elongation rate denotes the difference in the length of the plant’s above-ground part before and after treatment. The equation for calculating the plant elongation rate is as follows (Equation (1)):

Plant elongation rate (%) = [(PH_2_ − PH_1_)/PH_1_] × 100% (1)

where *PH_1_* and *PH_2_* indicate the above-ground heights before and after treatment, respectively.

The dry weight was the measured weight of the above-ground part of each plant following full-submergence treatment for 0, two, four, six, and eight days. Tissues (whose weight was equal to the fresh weight) were dried in an oven at 60 °C for two days. The weight of each plant, which represented the dry weight, was then measured. In each independent experiment, the mean dry weights of at least 30 plants were measured. The experiments were repeated more than three times.

To measure the chlorophyll content, nine-day-old seedlings were subjected to full-submergence treatment for eight days and a recovery period of seven days. The above-ground parts (30 mg) of the rice seedlings were ground with liquid nitrogen. A total of 2 mL of sodium phosphate buffer (50 mM, pH 6.8) was added to the ground seedlings. A total of 960 μL of 99% ethanol was blended into 40 μL of the extract. The mixture was evenly blended in a 1.5-mL microcentrifuge tube (Eppendorf tube) and placed at 4 °C in darkness for 30 min, followed by 1000× g centrifugation at 4 °C for 15 min. The absorbance of the supernatant at OD.665 and 649 was determined with a spectrophotometer (Metertec SP8001). The blank was 99% ethanol. More than three independent trials were conducted. The equations for calculating the chlorophyll content are as follows (Equations (2)–(4)):

Chlorophyll a = (13.7 × A_665_) − (5.76 × A_649_) [μgChL (40 μL)^−1^] (2)

Chlorophyll b = (25.8 × A_649_) − (7.6 × A_665_) [μgChL (40 μL)^−1^] (3)

Total chlorophyll = (6.1 × A_665_) + (20.04 × A_649_) [μgChL (40 μL)^−1^] (4)

### 5.3. Histochemical Staining and Antioxidative Enzyme Activity Assay

The accumulation of H_2_O_2_ in cells was visualized by 3, 3′-diaminobenzidine staining as previously described [29]. The experiments were repeated three times. Protein quantitation of the samples were performed after their extraction by using the Bradford protein assay in order to determine the antioxidant enzyme activity [30]. The above-ground parts of rice were ground into powder with liquid nitrogen and analyzed according to the method modify from Kar and Mishra to determine the CAT activity [31]. A total of 4 mL of 50 mM sodium phosphate buffer (pH 6.8) was added in the pre-chilled mortar for performing homogeneous grinding. The mixture was then subjected to centrifugation at 12,000× g and 4 °C for 20 min. The obtained supernatant was the enzyme extract. Protein quantitation was performed on the enzyme extract by using the Bradford assay. Enzyme extract with a volume corresponding to 1 mg of protein and 100 mM of sodium phosphate buffer (pH 7.0) (the total volume of the extract and buffer was 2.9 mL) were evenly blended with 100 μL of 1 M H_2_O_2_. A spectrophotometer (Metertec SP8001) was used at a wavelength of 240 nm to continuously detect the change in absorbance during a five-minute period. The detection interval was 10 s, and 99% ethanol was used as the blank. The unit of enzyme activity was μmol of H_2_O_2_ consumed per minute. The above-ground parts of rice were ground into powder with liquid nitrogen in a pre-chilled mortar and analyzed according to the method of MacAdam et al. to determine the total POD activity [32]. Lin and Kao [33] proposed that water-soluble and ionic-bonded peroxidase can be extracted by adding potassium chloride (KCl) in buffer solution. They defined the sum of the aforementioned two peroxidase types as the total activity. In the experiment, 4 mL of 50 mM potassium phosphate buffer (pH 5.8), which included 0.8 M KCl, was added in a pre-chilled mortar for obtaining homogeneous powder. The mixture was then subjected to centrifugation at 12,000× g and 4 °C; for 20 min. The obtained supernatant was the enzyme extract. Protein quantitation was performed on the extract by using the Bradford assay. Enzyme extract with a volume corresponding to 1 mg of protein and 50 mM of potassium phosphate buffer (pH 5.8) were homogeneously blended with 1 mL of 21.6 mM guaiacol and 0.9 mL of 39 mM H_2_O_2_. A spectrophotometer (MetertecSP8001) was used at a wavelength of 470 nm to continuously detect the changes in the absorbance during a 30-s period. The detection interval was 10 s and 99% ethanol was used as the blank. The unit of enzyme activity was μmol of tetra guaiacol produced per minute.

### 5.4. Quantitative RT-PCR Analyses

The rice samples were placed in a mortar and ground into powder using liquid nitrogen. A total of 2 mL of TRIZOL reagent (Roche Applied Science, Penzberg, Upper Bavaria, Germany) was added to the mortar for extracting the total RNA. The total RNA extracted was then treated using TURBO DNase (Ambion, Austin, TX, USA). A Nano Drop Lite ultra-micro spectrophotometer (Thermo Scientific, Waltham, MA, USA) was used in order to measure the optical density of 2 μL of RNA and examine its concentration and quality. An MMLV First-Strand Synthesis Kit (Gene Direx, Grand Island, NY, USA) was used to synthesize the first strand of cDNA [reverse transcription (RT)]. qRT-PCR was performed, as previously described [34] using a Bio-Rad CFX instrument (CFX Connect™, Bio-Rad, Hercules, CA, USA) with Power SYBR Green PCR Master Mix (Gene-Mark, Taipei, Taiwan), according to the manufacturer’s recommendations. The ubiquitin gene was used as an internal control for normalization. The relative expression levels were analyzed using Bio-Rad CFX Manager (version 3.1). The experiments were repeated three times independently with duplicate samples. Table 2 presents the primer sequences for qRT-PCR.

### 5.5. Statistical Analysis

In this experiment, Statistical Analysis System (SAS) software version 9.4 was used for statistical analysis. We used analysis of variance (ANOVA) for pre-comparison, and then used Duncan’s Multiple Range Test for multiple comparisons. The different alphabets indicate significant differences in the performance of the five rice varieties (*p* < 0.05). All of the tests were conducted in more than three independent tests.

## Figures and Tables

**Table 1:** The fresh weight, dry weight and ratio of dry mass in five japonica varieties during submergence stress.

Treatment *		Days	Fresh Weight (mg·plant^−1^)					Dry Weight (mg·plant^−1^)					Ratio of Dry Mass (%)				
	Variety **		0	2	4	6	8	0	2	4	6	8	0	2	4	6	8
CK	TN11		42.2 ± 0.1c ***	62.6 ± 1.3b	65.0 ± 2.0b	77.8 ± 0.2b	97.2 ± 0.5c	5.5 ± 0.2b	8.7 ± 0.2a	8.7 ± 0.4b	11.2 ± 0.2b	13.8 ± 0.2a	13.1 ± 0.1b	13.9 ± 0.1a	13.4 ± 0.2a	14.4 ± 0.3a	14.2 ± 0.1a
	TN14		41.4 ± 1.1c	58.5 ± 0.4c	63.0 ± 2.2b	73.0 ± 1.5c	82.8 ± 2.4d	4.7 ± 0.2c	8.2 ± 0.3b	8.5 ± 0.5b	10.1 ± 0.5c	10.3 ± 0.6c	11.4 ± 0.4c	14.0 ± 0.5a	13.4 ± 0.4a	13.9 ± 0.5ab	12.4 ± 0.4c
	KH139		47.6 ± 0.7a	69.0 ± 1.2a	72.2 ± 1.9a	86.7 ± 1.9a	106.7 ± 2.1a	7.0 ± 0.3a	8.8 ± 0.2a	10.7 ± 0.7a	12.5 ± 0.3a	13.7 ± 0.3a	14.7 ± 0.4a	12.7 ± 0.1b	14.9 ± 0.8b	14.3 ± 0.6a	12.8 ± 0.5c
	TK9		42.4 ± 1.2b	58.0 ± 2.6c	65.4 ± 4.1b	83.5 ± 3.2a	101.5 ± 1.1b	5.6 ± 0.2b	7.8 ± 0.2bc	9.2 ± 0.2b	11.4 ± 0.4b	12.6 ± 0.2b	13.3 ± 0.5b	13.4 ± 0.2ab	14.0 ± 1.1ab	13.7 ± 0.9ab	12.4 ± 0.3c
	TNG71		44.8 ± 0.9c	67.2 ± 1.4a	72.7 ± 3.1a	86.4 ± 2.0a	103.4 ± 1.4b	5.3 ± 0.3b	7.5 ± 0.5c	8.9 ± 0.1b	11.4 ± 0.2b	14.0 ± 0.3a	11.9 ± 0.8c	11.2 ± 0.9c	12.2 ± 0.6c	13.2 ± 0.3b	13.5 ± 0.4b
SUB	TN11		42.9 ± 1.0b	64.0 ± 1.4b	82.4 ± 2.4b	104.7 ± 2.1b	100.3 ± 1.1d	5.6 ± 0.3bc	7.5 ± 0.2b	9.7 ± 0.6a	10.2 ± 0.4ab	10.3 ± 0.4b	13.0 ± 0.3ab	11.7 ± 0.4a	11.7 ± 0.5a	9.8 ± 0.5a	10.3 ± 0.3ab
	TN14		38.4 ± 1.4c	63.4 ± 0.9b	72.1 ± 1.3c	93.9 ± 3.6c	79.8 ± 2.5e	5.2 ± 0.1c	6.5 ± 0.5c	7.3 ± 0.3b	8.4 ± 0.8c	8.7 ± 0.6c	13.4 ± 0.5ab	10.2 ± 0.8b	10.1 ± 0.5bc	9.0 ± 0.9a	10.9 ± 0.7a
	KH139		47.9 ± 1.0a	71.9 ± 1.2a	93.5 ± 3.0a	119.1 ± 1.9a	118.6 ± 1.4b	6.5 ± 0.4a	7.6 ± 0.5ab	9.1 ± 0.2a	10.6 ± 0.7a	11.2 ± 0.6a	13.5 ± 0.4a	10.5 ± 0.9b	9.8 ± 0.1c	8.9 ± 0.5a	9.4 ± 0.5b
	TK9		42.5 ± 1.4b	58.9 ± 1.8c	83.8 ± 3.6b	103.9 ± 3.9b	107.2 ± 5.7c	5.6 ± 0.2b	6.2 ± 0.3c	9.4 ± 0.5a	9.4 ± 0.4bc	10.2 ± 0.3b	13.3 ± 0.7ab	10.5 ± 0.3b	11.3 ± 0.3a	9.0 ± 0.6a	9.6 ± 0.8b
	TNG71		43.8 ± 2.1b	69.8 ± 1.2a	92.8 ± 0.7a	113.9 ± 3.2a	126.1 ± 3.2a	5.4 ± 0.2bc	8.2 ± 0.3a	9.8 ± 0.1a	10.1 ± 0.2ab	10.5 ± 0.2ab	12.4 ± 0.7b	11.8 ± 0.4a	10.5 ± 0.1b	8.9 ± 0.3a	8.3 ± 0.3c

* CK (control check) was the control group; SUB (submergence) was the full-submergence treatment group. ** Variety: TN11; Tainan 11, TN14; Tainan 14, KH139; Kaohsiung 139, TK9; Taiken 9, TNG71; Tainung 71. *** Statistical Analysis System (SAS) 9.4 was used to conduct Duncan’s analysis. The different alphabets indicate the significant differences in the number of treatment days and performance among the five rice varieties (*p* < 0.05). Each treatment involved at least 30 plants, and independent experiments were repeated least three times.

**Table 2:** Primers used for quantitative RT-PCR experiments.

Gene Name	Primer Sequence
*OsUbiquitin—*forward	5’-aaccagctgaggcccaaga-3’
*OsUbiquitin—*reverse	5’-acgattgatttaaccagtccatga-3’
*OsSUS1—*forward	5’-catctcaggctgagactctga-3’
*OsSUS1—*reverse	5’-caaattcaatcgaccttactt-3’
*OsADH1—*forward	5’-gcaaatttctggctttgtcaatcagta-3’
*OsADH1—*reverse	5’-cgccaaaagatcactgattcttaacaa-3’

## References

[1] Food and Agriculture Organization of the UN, International Fund for Agricultural Development, UNICEF, World Food Programme, WHO (2018). The State of Food Security and Nutrition in the World.

[2] Boyland M. (2015). In pursuit of effective flood risk management in the Mekong region. Focus.

[3] Hirose T., Scofield G.N., Terao T. (2008). An expression analysis profile for the entire sucrose synthase gene family in rice. Plant Sci..

[4] Bailey-Serres J., Voesenek L.A.C.J. (2008). Flooding stress: Acclimations and genetic diversity. Annu. Rev. Plant Biol..

[5] Fukao T., Xu K., Ronald P.C., Bailey-Serres J. (2006). A variable cluster of ethylene response factor-like genes regulates metabolic and developmental acclimation responses to submergence in rice. Plant Cell.

[6] Subbaiah C.C., Palaniappan A., Duncan K., Rhoads D.M., Huber S.C., Sachs M.M. (2006). Mitochondrial localization and putative signaling function of sucrose synthase in maize. J. Biol. Chem..

[7] Loreti E., Valeri M.C., Novi G., Perata P. (2018). Gene regulation and survival under hypoxia requires starch availability and metabolism. Plant Physiol..

[8] Shabala S., Munns R., Shabala S. (2012). Salinity Stress: Physiological Constraints and Adaptive Mechanisms. Plant Stress Physiology.

[9] Loreti E., van Veen H., Perata P. (2016). Plant responses to flooding stress. Curr. Opin. Plant Biol..

[10] Garnczarska M., Bednarski W. (2004). Effect of a short-term hypoxic treatment followed by re-aeration on free radicals level and antioxidative enzymes in lupine roots. Plant Physiol. Biochem..

[11] Geigenberger P. (2003). Response of plant metabolism to too little oxygen. Curr. Opin. Plant Biol..

[12] Vergara R., Parada F., Rubio S., Pérez F.J. (2012). Hypoxia induces H_2_O_2_ production and activates antioxidant defence system in grapevine buds through mediation of H_2_O_2_ and ethylene. J. Exp. Botany.

[13] Chapman J.M., Muhlemann J.K., Gayomba S.R., Muday G.K. (2019). RBOH-dependent ROS synthesis and ROS scavenging by plant specialized metabolites to modulate plant development and stress responses. Chem. Res. Toxicol..

[14] Fukao T., Bailey-Serres J. (2008). Submergence tolerance conferred by Sub1A is mediated by SLR1 and SLRL1 restriction of gibberellin responses in rice. Proc. Natl. Acad. Sci. USA.

[15] Hattori Y., Nagai K., Ashikari M. (2011). Rice growth adapting to deep water. Curr. Opin. Plant Biol..

[16] Farooq M., Siddique K.H.M., Rehman H., Aziz T., Lee D.J., Wahid A. (2011). Rice direct seeding: Experiences, challenges and opportunities. Soil Tillage Res..

[17] Kashiwagi J., Hamada K., Jitsuyama Y. (2018). Rice (*Oryza sativa* L.) germplasm with better seedling emergence under direct sowing in flooded paddy field. Plant Genet. Resour. Charact. Util..

[18] Panda D., Sarkar R.K. (2012). Leaf photosynthetic activity and antioxidant defense associated with Sub1 QTL in rice subjected to submergence and subsequent re-aeration. Rice Sci..

[19] Matile P., Hortensteiner S., Thomas H., Krautler B. (1996). Chlorophyll breakdown in senescent leaves. Plant Physiol..

[20] Sone C., Sakagami J.I. (2017). Physiological mechanism of chlorophyll breakdown for leaves under complete submergence in rice. Crop Sci..

[21] Blokhina O., Virolainen E., Fagerstedt K.V. (2003). Antioxidants, oxidative damage and oxygen deprivation stress: A review. Ann. Bot..

[22] Anjum S.A., Xie X., Wang L., Saleem M., Man C., Lei W. (2011). Morphological, physiological and biochemical responses of plants to drought stress. Afr. J. Agric. Res..

[23] Gill S.S., Tuteja N. (2010). Reactive oxygen species and antioxidant machinery in abiotic stress tolerance in crop plants. Plant Physiol. Biochem..

[24] Ali S., Huang Z., Li H.X., Bashir M.H., Ren S.X. (2013). Antioxidant enzyme influences germination, stress tolerance, and virulence of *Isaria Fumosorosea*. J. Basic Microbiol..

[25] Liu J., Sun X., Xu F., Zhang Y., Zhang Q., Miao R., Zhang J., Liang J., Xu W. (2018). Suppression of *OsMDHAR4* enhances heat tolerance by mediating H_2_O_2_-induced stomatal closure in rice plants. Rice.

[26] Bailey-Serres J., Fukao T., Ronald P., Ismail A., Heuer S., Mackill D. (2010). Submergence tolerant rice: SUB1’s journey from landrace to modern cultivar. Rice.

[27] Yoshida S., Forno D.A., Cock J.H., Gomez K.A. (1976). Routine Procedure for Growing Rice Plants in Culture solution. Laboratory Manual for Physiological Studies of Rice.

[28] Zhu G.L., Chen Y.T., Ella E.S., Ismail A.M. (2019). Mechanisms associated with tiller suppression under stagnant flooding in rice. J. Agron. Crop. Sci..

[29] Huang Y., Yeh T., Yang C. (2019). Ethylene signaling involves in seeds germination upon submergence and antioxidant response elicited confers submergence tolerance to rice seedlings. Rice.

[30] Bradford M.M. (1976). A rapid and sensitive method for the quantitation of microgram quantities of protein utilizing the principle of protein-dye binding. Anal. Biochem..

[31] Kar M., Mishra D. (1976). Catalase, peroxidase and polyphenol oxidase activities during rice leaf senescence. Plant Physiol..

[32] MacAdam J.W., Nelson C.J., Sharp R.E. (1992). Peroxidase activity in the leaf elongation zone of tall fescue1. Plant Physiol..

[33] Lin C.C., Kao C.H. (1999). NaCl induced changes in ionically bound peroxidase activity in roots of rice seedlings. Plant Soil.

[34] Yang S.Y., Wu Y.S., Chen C.T., Lai M.H., Yen H.M., Yang C.Y. (2017). Physiological and molecular responses of seedlings of an upland rice (‘Tung Lu 3’) to total submergence compared to those of a submergence-tolerant lowland rice (‘FR13A’). Rice.

